# Dr. Thomas J. Thurber

**Published:** 1916-06

**Authors:** 


**Dr. Thomas J. Thurber,** N. Y. Homoeopathic 1883, died at his home in Rochester, Mch. 25, aged 72.

